# Diagnostic Performance of the Strength‐Duration Test for Bedside Screening of Critical Illness Polyneuropathy and/or Myopathy: A Prospective, Cross‐Sectional Study

**DOI:** 10.1002/mus.70368

**Published:** 2026-07-30

**Authors:** José Roberto de Deus Macedo, Emerson Fachin‐Martins, Marilia Mendes Rodrigues, Marta Rodrigues de Carvalho, Rubens Nelson Morato Fernandez, Paulo Eugênio Silva

**Affiliations:** ^1^ Health Sciences and Technologies PhD Program, Faculdade de Ciências e Tecnologias Em Saúde, Campus UnB Ceilândia Universidade de Brasília Brasília Brazil; ^2^ Intensive Care Division, Hospital de Base do Distrito Federal Instituto de Gestão Estratégica de Saúde do Distrito Federal Brasília Brazil; ^3^ BEM‐TE‐VI Tech Platform, Research and Tech Park Universidade de Brasília Brasília Brazil; ^4^ Physical Therapy Division, Hospital de Base do Distrito Federal Instituto de Gestão Estratégica de Saúde do Distrito Federal Brasília Brazil; ^5^ Neurophysiology Division, Hospital de Base do Distrito Federal Instituto de Gestão Estratégica de Saúde do Distrito Federal Brasília Brazil; ^6^ Physical Rehabilitation Division, Hospital de Base do Distrito Federal Instituto de Gestão Estratégica de Saúde do Distrito Federal Brasília Brazil; ^7^ Faculty of Medicine, Physical Rehabilitation Division University of São Paulo São Paulo Brazil

**Keywords:** chronaxie, critical illness polyneuropathy, sensitivity, specificity, strength‐duration test

## Abstract

**Introduction/Aims:**

Critical illness polyneuropathy and/or myopathy (CIP/CIM) is a major cause of weakness in the intensive care unit (ICU). The availability of conventional electrodiagnostic testing may be limited. Alternative electrophysiologic methods, including the strength‐duration test (SDT) and the stimulus electrodiagnosis test (SET), have been proposed as bedside screening adjuncts. This study aimed to evaluate the SDT's diagnostic metrics for CIP/CIM screening and to compare with the SET's performance.

**Methods:**

This prospective, cross‐sectional study included adult, mechanically ventilated ICU patients. Single assessment per patient comprised the peroneal simplified electrophysiological test (PENT), SDT, and SET. Using PENT as the reference test, we calculated the accuracy, sensitivity, specificity, predictive values, and likelihood ratios of SDT. Receiver operating characteristic curves were constructed to assess the discriminative performance and optimal cutoff.

**Results:**

Sixty participants were enrolled, yielding 101 assessments, including bilateral evaluations when feasible. The SDT demonstrated an area under the curve (AUC) of 0.8 (95% CI: 0.7–0.8, *p* < 0.001), with an optimal cutoff of 600 mC. At this cutoff, SDT showed a sensitivity of 73% (95% CI: 61–82), specificity of 68% (95% CI: 53–81), accuracy of 71% (95% CI: 62–79), and positive likelihood ratio of 2.3 (95% CI: 1.41–3.78) for screening probable CIP/CIM. The SET yielded an AUC of 0.7 (95% CI: 0.6–0.8, *p* = 0.001).

**Discussion:**

The SDT showed potential as a bedside screening adjunct for CIP/CIM in the ICU setting. Further multicenter studies are required to validate these findings and to define the role of SDT in clinical practice.

**Abbreviations**
AUC: area under curve
BMI: body mass index
CIM: critical illness myopathy
CIP: critical illness polyneuropathy
CIP/CIM: critical illness polyneuropathy and/or myopathy
CMAP: compound muscle action potential
ICU: intensive care unit
ICUAW: intensive care unit‐acquired weakness
MV: mechanical ventilation
n‐EMG: needle‐electromyography
PENT: peroneal simplified electrophysiological test
RASS: Richmond agitation‐sedation scale
ROC: receiver operating characteristic
SAPS 3: simplified acute physiology score 3
SDT: strength‐duration test
SET: stimulus electrodiagnosis test
SOFA: sequential organ failure assessment

## 1 Introduction

Critical illness polyneuropathy (CIP) and critical illness myopathy (CIM) are common neuromuscular complications of critical illness and major contributors to intensive care unit‐acquired weakness (ICUAW) [1, 2]. CIP is a diffuse sensorimotor axonopathy that affects 30%–50% of critically ill patients, with electrophysiological abnormalities reported in about 70% of patients with sepsis [3, 4]. CIM is a primary myopathy characterized by altered muscle excitability, and it also occurs frequently in ICU populations [5]. CIP and CIM frequently co‐occur and present with similar clinical features, so that they are designated critical illness polyneuropathy and/or myopathy (CIP/CIM) when they are not distinguished—a term also used for screening purposes [6]. CIP/CIM has been associated with difficulty weaning from the ventilator [7], persistent limb weakness [8], and worse long‐term outcomes [9, 10].

The diagnosis of ICUAW begins with clinical assessment and may be supplemented by electrophysiological studies when indicated [11, 12]. In deeply sedated or uncooperative patients, simplified approaches such as the peroneal simplified electrophysiological test (PENT) may help identify probable CIP/CIM because of their high reported sensitivity, although their specificity is more limited and they do not provide definitive etiologic classification [13, 14, 15]. Regardless of the diagnostic definition, early detection of an electrophysiological neuromuscular disorder (e.g., reduced CMAP) may predict functional outcome [16] and survival [17] in critically ill patients, even without the development of ICUAW (absence of weakness). Within this context, bedside methods that quantify neuromuscular excitability may be explored as adjunct screening approaches rather than as substitutes for established diagnostic strategies.

The stimulus electrodiagnosis test (SET) [18, 19] and the strength‐duration test (SDT) [20] are based on established electrophysiological principles, including rheobase, chronaxie, and the strength duration relationship. SET provides point estimates of excitability, whereas SDT evaluates responses across multiple pulse durations and may therefore offer a broader description of the strength duration relationship [21]. Both approaches have been investigated in peripheral neuromuscular disorders [19] and more recently, in critically ill patients [20, 21, 22], but their diagnostic metrics for bedside screening of probable CIP/CIM remain insufficiently defined.

The present study aimed to evaluate the diagnostic metrics of the SDT for bedside screening of probable CIP/CIM, and to compare its performance with that of SET, using PENT as the reference test.

## 2 Methods

### 2.1 Study Design

This prospective, cross‐sectional diagnostic accuracy study was conducted in critically ill adult patients from April 2024 to June 2025.

### 2.2 Participants

The study population comprised critically ill adult patients (aged ≥ 18 years, of both sexes) who had been sedated for at least 3 days, were receiving mechanical ventilation, and had no history of pre‐existing neuromuscular disease or orthopedic deformity that could interfere with lower‐limb testing. Patients were excluded if they had bilateral lower‐limb conditions precluding nerve conduction studies, such as pitting edema graded as 3 or 4 on a 4‐point scale, fractures, amputation, plaster casts, or local skin injury; a body mass index > 35 kg/m^2^; brain death; pregnancy; current use of neuromuscular blockers; or corticosteroid use from ICU admission to electrodiagnostic recording.

The study was conducted in the critical care units of a tertiary public reference hospital in the Federal District, Brazil. The protocol complied with the Declaration of Helsinki and was approved by the local ethics committee (HB/IGESDF, Brasília, DF, Brazil; approval number 5.731.715). Written informed consent was obtained from the legally authorized representative of each participant because all participants were intubated and sedated at enrollment. Consecutive recruitment was performed after eligibility confirmation until the prespecified sample size was reached.

### 2.3 Test Methods

Each participant underwent a single study session during the ICU stay. PENT, SET, and SDT were applied in randomized order to the lower limbs, bilaterally whenever feasible. When only one limb could be assessed because of local contraindications or practical limitations, that limb contributed one assessment. Skin surface temperature was recorded before testing and maintained above 32°C using an infrared thermometer (Easy Sensor G‐TECH, Joytech Healthcare Co., China). The PENT, a simplified motor nerve conduction study with high reported sensitivity for CIP/CIM, was selected as the reference test because comprehensive nerve conduction studies and needle‐electromyography (n‐EMG) were not feasible in this deeply sedated cohort. The SDT was the index test, and the SET was included as a comparator. This diagnostic‐accuracy study was reported according to STARD 2015 [23].

### 2.4 Outcomes

The primary outcome was the diagnostic performance of peroneal SDT electrical charge for bedside screening of probable CIP/CIM, including the definition of an optimal cutoff point.

Secondary outcomes included comparison of the diagnostic metrics of SDT and SET for screening of probable CIP/CIM, using PENT as the reference test. Safety and feasibility of SET and SDT were also assessed. Safety monitoring included continuous inspection for local hyperemia, burns, or other adverse skin effects during and immediately after testing. The protocol was interrupted if any clinically relevant skin change was observed. Feasibility was assessed according to test completion, and the time required to perform SDT.

### 2.5 Variables

After enrollment, baseline variables were collected to characterize the clinical profile and to describe illness severity, sedation depth, and organ dysfunction in the population in which the tests were applied. These variables included Simplified Acute Physiology Score 3 (SAPS 3) [24], Sequential Organ Failure Assessment (SOFA) score [24], Richmond Agitation‐Sedation Scale (RASS) [25], and the presence of sepsis or septic shock [26].

All participants underwent assessment with a 30‐min interval between tests. Examinations were performed by two independent examiners: Examiner 1 (JRDM) performed the PENT, whereas Examiner 2 (PES) performed the SET and SDT. The examiners remained blinded to each other's results and diagnostic impressions throughout data collection.

Previous studies have reported PENT sensitivity ranging from 92% to 100%, although its specificity is lower than that of comprehensive electrodiagnostic testing [15]. PENT was performed with a portable electroneurograph (Neuro‐MEP‐Micro, Neurosoft, Russia). The low‐pass filter was set at 3 Hz and the high‐pass filter at 10 kHz. Device sensitivity and sweep speed were set at 3 mV and 2 ms, respectively. The portable electroneurograph had previously been validated by our Neurophysiology Department through paired testing that yielded results comparable to those of the service standard system (Nihon Kohden Neuropack M1 MEB‐9200, Tokyo, Japan), which had been standardized locally according to Katirji [27] and Tan [28] reference criteria.

Stimulation was performed at two sites to record distal motor latency, CMAP amplitude, and conduction velocity for each peroneal nerve. Amplitude was measured from baseline to the negative peak. Surface recording electrodes (Ambu, WhiteSensor 0315M, Ambu, Denmark) were placed over the belly and tendon of the extensor digitorum brevis. The peroneal nerve was stimulated at the anterior ankle, slightly lateral to the tibialis anterior tendon, 6–8 cm from the recording electrodes and below the fibular head for conduction‐velocity recording. Incremental electrical stimulation was delivered until a supramaximal CMAP was obtained. All assessments were performed with the patient in the supine position, with 30° head‐of‐bed elevation and the lower limbs extended.

### 2.6 Diagnostic Criteria for CIP/CIM

CMAP amplitudes below 80% [14, 15] of Katirji's normative values were considered abnormal, defined as < 3 mV for ages 20–50 years and < 2.5 mV for ages 51–90 years [27]. Axonopathy and/or myopathy patterns in motor conduction velocity were assessed according to Tan's criteria: at least 80% of the lower limit of normal if CMAP was < 50% of normal, or at least 90% of the lower limit of normal if CMAP was > 50% of normal [28]. For this study, probable CIP/CIM was defined separately for each limb analyzed, as reduced CMAP amplitude with normal or only mildly reduced conduction velocity [3].

### 2.7 Stimulus Electrodiagnosis Test and Strength‐Duration Test

SET and SDT were performed using a bedside peripheral electrical neuromodulator (Recare, Visuri, Brazil). The device had been recalibrated by the manufacturer and previously standardized in an earlier study [21]. Both tests were used to assess neuromuscular excitability of the extensor digitorum brevis in response to electrical stimulation delivered to elicit the smallest visually detectable contraction. Two 3‐cm‐diameter circular electrodes (ValuTrode, Axelgaard, Denmark) were placed over the dorsum of the foot near the ankle for peroneal nerve stimulation, following the recommendations of Latronico et al. [13] All assessments were performed with the patient in the supine position, with 30° head‐of‐bed elevation and the lower limbs extended.

### 2.8 SET

During SET, neuromuscular excitability was quantified by measuring rheobase and chronaxie. Rheobase was defined as the minimum current intensity required to elicit excitation with a long rectangular pulse (> 200 ms). Chronaxie was defined as the shortest pulse duration required to reach excitation using a current amplitude equal to twice the rheobase. Rheobase and chronaxie were measured with a single rectangular, biphasic, symmetric pulse. For rheobase assessment, current amplitude was increased from 1 to 240 mAp‐p in 1 mAp‐p increments until a minimal visible contraction was observed, using a pulse duration of 500 ms and an inter‐pulse interval of 2 s. Therefore, the pulse was delivered every 3 s, corresponding to a stimulation frequency of approximately 0.33 Hz. For chronaxie assessment, current amplitude was set at twice the rheobase value, and pulse duration was then increased from 0.1 ms steps to 1 ms. After 1 ms, the pulse duration was increased in 1‐ms steps until chronaxie was reached—a minimal visible contraction usually below 10 ms [21, 22]. Based on previous publications, chronaxie value > 1 ms was prespecified as SET threshold [19].

### 2.9 SDT

The SDT used a single rectangular, biphasic, symmetric electrical pulse delivered at the following durations: 0.1, 0.2, 0.5, 1, 2, 5, 10, 20, and 50 ms. For this pulse configuration, pulse duration was defined as the sum of the positive‐ and negative‐phase widths. For each pulse duration, the examiner adjusted current intensity from 1 to 240 mAp‐p to elicit a minimal visible contraction. Electrical charge during SDT was automatically calculated by the Recare software as the product of pulse amplitude (mAp‐p) and pulse duration (ms) and was expressed in millicoulombs (mC). Higher charge values were interpreted as reflecting lower neuromuscular excitability. Because no validated ICU‐specific cutoff was available for SDT electrical charge at this anatomical site, the positivity threshold was defined exploratorily from the ROC analysis.

### 2.10 Study Size

The sample size was calculated with Power Analysis and Sample Size (PASS) software according to the recommendations of Flahault et al. [29]. The calculation included disease prevalence, the null hypothesis, statistical power (1‐β), alpha level (α), maximum acceptable distance from sensitivity (δ), and the expected sensitivity and specificity. Prevalence of neurophysiological abnormalities was estimated at 55% from a previous study. Statistical power and alpha were set at 80% and 5%, respectively, and δ was set at 25%. Expected sensitivity was set at 80% based on Paternostro‐Sluga et al., whereas specificity was conservatively set at 70% because directly comparable data were unavailable. After accounting for an anticipated 5% drop‐out rate, at least 100 limb assessments were required.

### 2.11 Statistical Methods

Data normality was assessed with the Shapiro–Wilk test. Non‐parametric variables are described as medians with interquartile ranges [IQR], and categorical variables are presented as absolute frequencies and percentages. Because PENT, SET, and SDT were recorded separately for each lower limb when feasible, diagnostic analyses were performed at the limb‐assessment level.

Receiver operating characteristic (ROC) curves were constructed for SDT and SET and compared with the DeLong test.

The optimal SDT cutoff point was determined with Youden's J index. Sensitivity, specificity, accuracy, positive and negative predictive values, and positive and negative likelihood ratios were calculated for the selected cutoff point with 95% confidence intervals estimated by Wilson's method. A *p*‐value < 0.05 was considered statistically significant. Statistical analyses were performed with IBM SPSS Statistics version 29 (IBM Corp., Armonk, NY) and R Statistical Software version 4.2.2 (R Core Team, Vienna, Austria).

## 3 Results

**FIGURE 1:**
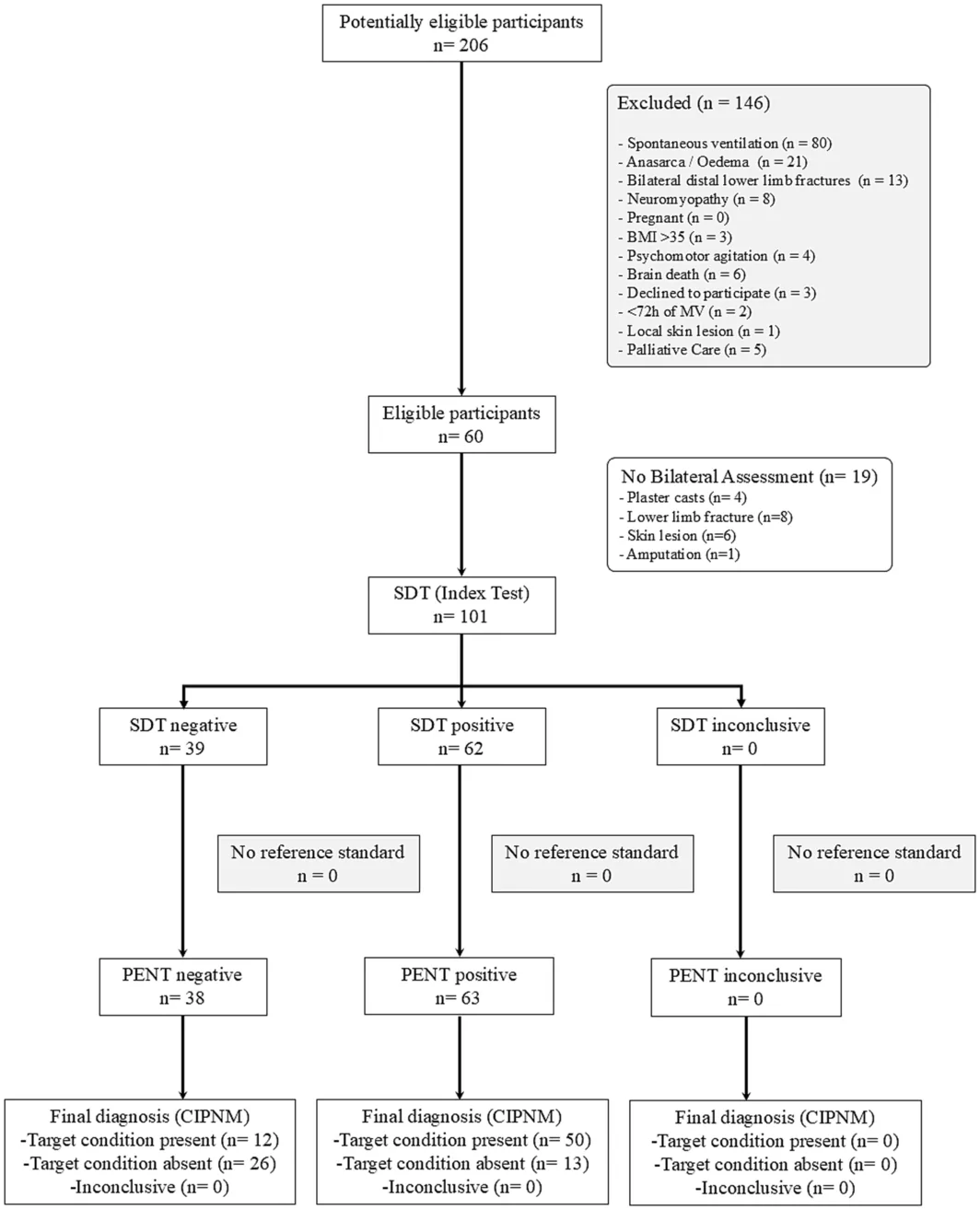
Flow diagram. The flow diagram was developed according to the Standards for Reporting of Diagnostic Accuracy (STARD). Assessments were performed bilaterally when feasible. The Strength‐Duration Test (SDT) was employed as the index test, with the Peroneal Simplified Electrophysiological Test (PENT) serving as the reference standard for diagnosing probable critical illness polyneuropathy and/or myopathy (CIP/CIM), which was defined as the target condition.

A total of 206 participants were screened, and 60 (29.1%) were enrolled. These participants yielded 101 limb assessments. Further details regarding participant flow are provided in the flow diagram (Figure 1).

Baseline characteristics are summarized in Table 1.

**TABLE 1:** Baseline demographic and clinical characteristics.

Variables	Participants (*n* = 60)	Negative PENT (*n* = 17)	Positive PENT (*n* = 43)
Age, years	47 [34.3–57.8]	40 [28.5–50.5]	49 [35–59]
Sex, *n* (%) male	41 (68.3%)	12 (70.6%)	29 (67.4%)
SAPS 3, on admission	58 [49–67]	50 [48–67]	58 [51–67]
SOFA, on assessment	7 [6–9]	9 [6–10]	7 [6–9]
Ventilation days, on assessment	6 [4–10]	4 [3.5–8]	7 [5–12]
ICU length of stay (days), on assessment	4 [2–8.8]	3 [2–6.5]	5 [2–10]
Hospital length of stay (days), on assessment	6.5 [4–12]	5 [4–9]	8 [5–14]
Sepsis, *n* (%)	50 (83.3%)	14 (82.4%)	36 (83.7%)
Septic Shock, *n* (%)	24 (40%)	8 (47.5%)	16 (37.2%)
RASS score, on assessment	−5 [−5–−5]	−5 [−5–−5]	−5 [−5–−5]
Norepinephrine *n* (%)	24 (40%)	8 (47.5%)	16 (37.2%)

*Note:* PENT was considered “positive” when suggestive of CIP/CIM. Data are presented as count (percentage, %) or median [interquartile range, IQR].

Abbreviations: CIP/CIM, critical illness polyneuromyopathy and/or myopathy; ICU, intensive Care unit; PENT, peroneal simplified electrophysiological test; RASS, Richmond Agitation‐Sedation Scale; SAPS3, simplified acute physiology score 3; SOFA, sequential organ failure assessment.

**FIGURE 2:**
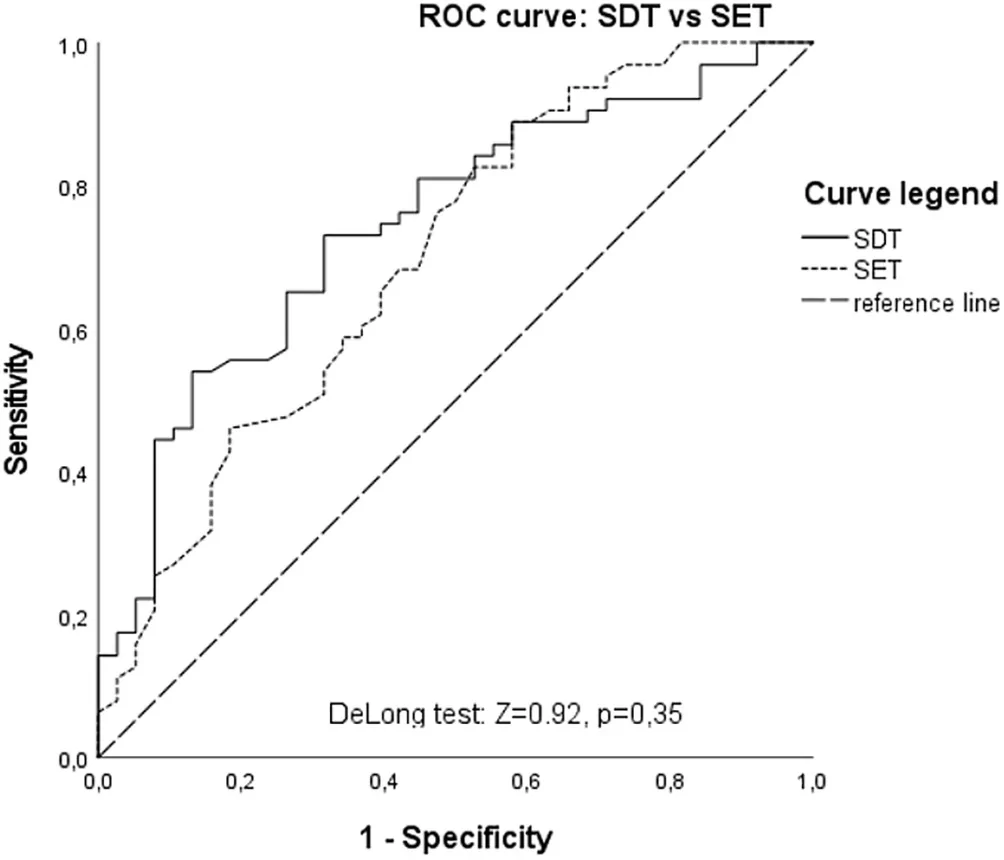
Receiver operating characteristic curves illustrating the discriminative power of SET and SDT for detecting probable CIP/CIM. For the SDT, the optimal cutoff point was identified at 600 mC (indicated by the black line representing its ROC curve), demonstrating an AUC of 0.8 (95% CI: 0.7–0.8, *p* < 0.001). The SET's ROC curve (traced line) yielded an AUC of 0.7 (95% CI: 0.6–0.8, *p* = 0.001). ROC, receiver operating characteristic; SDT, strength‐duration test; SET, stimulus electrodiagnosis test.

The ROC curves of SDT and SET did not differ significantly (Figure 2).

Exploratory analysis identified 600 mC as the optimal cutoff for SDT. The diagnostic metrics for SDT at this cutoff are presented in Table 2.

**TABLE 2:** Diagnostic metrics from SET and SDT with PENT as reference test.

Diagnostic metrics	SET value (IC 95%)	SDT value (IC 95%)
Cutoff point	Chronaxie > 1 ms (pre‐defined)	EC > 600 mC (exploratory)
Sensitivity	95% (87%–98%)	73% (61%–82%)
Specificity	29% (17%–45%)	68% (53%–81%)
Accuracy	70% (61%–78%)	71% (62%–79%)
PPV	69% (59%–78%)	79% (67%–88%)
NPV	79% (52%–92%)	60% (46%–74%)
PLR	1.3 (1.09–1.65)	2.3 (1.41–3.78)
NLR	0.2 (0.05–0.55)	0.4 (0.25–0.62)

Abbreviations: EC, electrical charge; NLR, negative likelihood ratio; NPV, negative predictive value; PENT, peroneal simplified electrophysiological test; PLR, positive likelihood ratio; PPV, positive predictive value; SDT, strength‐duration test; SET, stimulus electrodiagnosis test.

**FIGURE 3:**
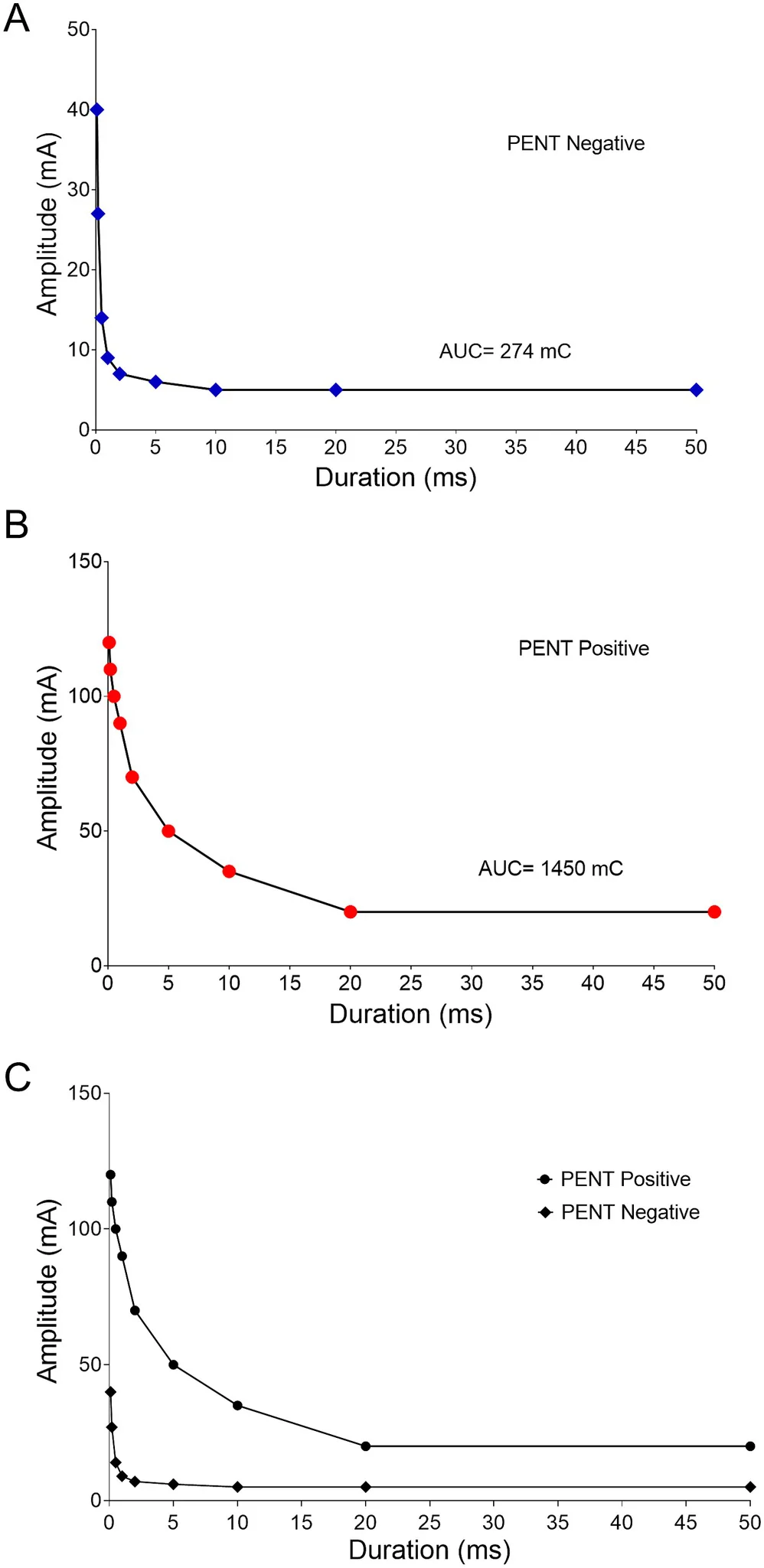
Representative examples of SDT electrical charges in PENT‐negative and PENT‐positive participants for probable CIP/CIM. This figure displays representative Strength‐Duration Test (SDT) curves, plotting pulse amplitude (mA) against pulse duration (ms), from individual participants. The shaded region beneath each curve represents the electrical charge, which is calculated as the area under the curve (AUC) derived from the product of pulse amplitude and pulse duration. (A) SDT curve from a participant with a negative PENT result, indicating no CIP/CIM. (B) SDT curve from a participant with a positive PENT result, indicating probable CIP/CIM. (C) Overlaid SDT curves from Panels A and B, illustrating the magnitude of the difference in electrical charge between participants with negative and positive PENT results. mA, milliampere; mC, millicoulomb; ms, milliseconds; PENT, peroneal simplified electrophysiological test; SDT, strength‐duration test.

Representative SDT curves according to PENT status are shown in Figure 3.

The diagnostic metrics for the SET, using the pre‐defined chronaxie cutoff of 1 ms, are presented in Table 2.

### 3.1 Safety and Feasibility

Sixty participants provided a maximum of 120 potential SET and SDT assessments, of which 101 were completed. Only 19 planned assessments (16%) were not performed in one lower limb because of clinical contraindications or practical limitations, as shown in Figure 1. No adverse events, including skin burns, were observed, and no examination was interrupted for safety reasons. The mean preparation and execution times were 8.1 ± 0.9 min for SET, 10.6 ± 1.4 min for SDT, and 6.2 ± 2.1 min for PENT.

## 4 Discussion

In this study, SDT showed promising discriminative performance for bedside screening of probable CIP/CIM and a more balanced sensitivity and specificity profile than SET. These findings suggest that SDT may be explored as an adjunct screening approach.

Paternostro‐Sluga et al. [19] proposed that SDT, by assessing the full strength‐duration relationship rather than an isolated chronaxie point, may better characterize neuromuscular excitability than SET. Our findings are consistent with that rationale and extend it to the peroneal nerve and extensor digitorum brevis in critically ill patients. In this study, excitability was summarized as electrical charge, as proposed by Silva et al. [21], which differs methodologically from previous SDT analyses and may partly explain between‐study differences [20].

Neither SDT nor SET was intended to establish a definitive diagnosis or to distinguish CIP from CIM. Rather, both tests were examined as simplified bedside screening approaches for probable CIP/CIM in a population in which full electrodiagnostic characterization was often impractical [3, 30]. This distinction is important because reduced PENT CMAP amplitudes may reflect axonopathy, myopathy, or both, and it may be a prognostic marker [16, 17]. Additional studies, such as sensory nerve conduction studies and n‐EMG, may be needed when etiologic discrimination is clinically relevant [5, 31].

In the present study, both tests were feasible and no local adverse events were observed. These findings support the technical tolerability of repeated stimulation under the study conditions. However, we do not propose SDT or SET as routine replacements for standard electrodiagnostic evaluation. Their potential role is more appropriately viewed as adjunct bedside tools, particularly in research settings or centers interested in simplified physiological screening protocols. Methodological standardization, especially regarding pulse‐duration ranges and equipment‐specific thresholds, remains necessary before broader adoption [20, 21].

Several limitations should be considered. First, the reference test was PENT rather than complete nerve conduction studies with n‐EMG or histopathology, and its limited specificity may have constrained the observed performance of the index tests. Second, the study did not differentiate CIP from CIM. Third, the post hoc derivation of the SDT cutoff may have produced optimistic estimates [23]. Fourth, both SET and SDT depended on visual identification of a minimal muscle twitch, which introduces some subjectivity despite standardized procedures [32, 33]. Finally, the cross‐sectional design did not allow longitudinal assessment.

Larger multicenter studies should validate the 600 mC cutoff and examine whether serial SDT measurements provide useful longitudinal information. Studies using more comprehensive electrodiagnostic reference standards should also clarify the relationship of SDT findings to neuropathic and myopathic components of ICU‐acquired weakness. Diagnostic algorithms combining SDT and SET also merit further evaluation.

Overall, SDT showed potential as a bedside screening approach for probable CIP/CIM in this study, but its clinical role remains to be defined in larger studies.

## Author Contributions

**Marta Rodrigues de Carvalho:** investigation, validation. **Emerson Fachin‐Martins:** supervision, project administration, funding acquisition, validation. **Marilia Mendes Rodrigues:** investigation, validation. **Rubens Nelson Morato Fernandez:** supervision, validation. **José Roberto de Deus Macedo:** investigation, writing – original draft, methodology, writing – review and editing, formal analysis, data curation, validation. **Paulo Eugênio Silva:** conceptualization, investigation, writing – original draft, writing – review and editing, formal analysis, data curation, validation.

## Funding

This work was supported by the FAPDF/Financeira de Serviços e Projetos (FINEP) (project number 00193‐00002152/2023‐95) enabled the acquisition of essential electromyography equipment, thereby facilitating the successful completion of this study.

## Ethics Statement

This study was conducted in accordance with the ethical principles outlined in the Declaration of Helsinki and was approved by the Institutional Review Board of HB/IGESDF, under protocol number 5.731.715.

## Consent

Written informed consent for publication was obtained.

## Conflicts of Interest

Author P.E.S. holds patents in neuromuscular electrical stimulation, has equity in Visuri SA, and serves as a scientific advisor for the company. The remaining authors declare no conflicts of interest.

## Data Availability

The data that support the findings of this study are available from the corresponding author upon reasonable request.
